# A Facile Approach to Improve Interchain Packing Order and Charge Mobilities by Self‐Assembly of Conjugated Polymers on Water

**DOI:** 10.1002/advs.201801497

**Published:** 2018-10-09

**Authors:** Yizhou Yang, Zitong Liu, Jianmei Chen, Zhengxu Cai, Zhijie Wang, Wei Chen, Guanxin Zhang, Xisha Zhang, Lifeng Chi, Deqing Zhang

**Affiliations:** ^1^ Beijing National Laboratory for Molecular Sciences CAS Key Laboratory of Organic Solids CAS Center of Excellence in Molecular Science Institute of Chemistry Chinese Academy of Sciences Beijing 100190 P. R. China; ^2^ School of Chemical Sciences University of Chinese Academy of Sciences Beijing 100049 P. R. China; ^3^ Institute of Functional Nano and Soft Materials (FUNSOM) Jiangsu Key Laboratory for Carbon‐Based Functional Materials and Devices Soochow University Suzhou 215123 China; ^4^ Beijing Key Laboratory of Construction Tailorable Advanced Functional Materials and Green Applications School of Material Science & Engineering Beijing Institute of Technology Beijing 100081 China; ^5^ Institute for Molecular Engineering and Materials Science Division Argonne National Laboratory 9700 Cass Avenue Lemont IL 60439 USA; ^6^ Institute for Molecular Engineering The University of Chicago 5640 South Ellis Avenue Chicago IL 60637 USA

**Keywords:** charge mobility, conjugated polymers, packing order, self‐assembly

## Abstract

Development of facile and economic approaches for assembling organic semiconductors into more ordered structures toward high charge mobilities is highly demanding for the fabrication of organic circuits. Here a simple and facile approach is reported to prepare conjugated polymer thin films with improved crystallinities and charge mobilities by self‐assembling semiconducting polymers on water. The formation of polymer thin films with more ordered structures is attributed to coffee ring effect induced by solvent‐evaporation on water, and the hydrophobic nature of conjugated polymers that forces the polymer chains to pack densely and orderly on water surface. This approach is applicable to typical semiconducting polymers, and charge mobilities of their thin films are boosted remarkably. Finally, this new method can be utilized to easily fabricate the array of field‐effect transistors with high charge mobilities in an economic way.

The past decades have witnessed the significant progresses in the development of organic and polymeric semiconductors with high charge mobilities.^1^, ^2^, ^3^, ^4^ Various conjugated molecules and polymers have been investigated intensively.^5^, ^6^, ^7^, ^8^, ^9^, ^10^, ^11^ These studies reveal that intermolecular/interchain packing and thin film crystallinity play a vital role in determining the charge transporting properties.^12^, ^13^ The most distinct advantage for polymeric semiconductors (conjugated polymers) is the solution processability. However, thin films of conjugated polymers are conventionally processed with spin‐coated technique and exhibit low crystallinities even after post‐treatments such as thermal annealing.^14^ Such thin films usually contain crystalline and amorphous domains.^15^ A few polymers such as poly(4,4,9,9‐tetrahexadecyl‐4,9‐dihydro‐s‐2,7‐indaceno[1,2‐b:5,6‐b′]dithiophene‐benzothiadiazole) (IDTBT)^16^ and Poly[*N*‐9′‐heptadecanyl‐2,7‐carbazole‐*alt*‐5,5‐(4′,7′‐di‐2‐thienyl‐2′,1′,3′‐benzothiadiazole)] (PCDTBT)^15^ with low crystallinity were reported to exhibit relatively high charge mobilities, which is attributed to the low energetic disorder of the polymeric backbones.^15^ However, more studies also manifest that thin films of conjugated polymers with more ordered structures show better charge transporting performances.^7^, ^8^, ^17^ Several approaches have been devised to improve the crystallinities of conjugated polymer chains and thus boost the charge mobilities by using zone casting,^17^ solution shearing,^18^ nanogrooved substrates,^19^ and capillary forces.^3^ Some of us have recently reported an efficient approach to improve the interchain packing order and thus enhance charge mobilities for conjugated D (donor)–A (acceptor) polymers by incorporation of tiny amounts of ionic additive in the thin films.^4^ It needs to point out that additional instruments or procedures are required for all these approaches.

Herein, we report a facile and effective approach to improve interchain packing order, thin film crystallinities and charge mobilities for conjugated polymers by solvent evaporation induced self‐***a***ssembly of conjugated polymers ***o***n ***w***ater, which is referred as to **AOW**. This **AOW** approach can be easily operated by dropping the conjugated polymer solutions onto the water surface, and the resulting thin films can be transferred to solid substrates easily. The resulting thin films of conjugated polymers show ordered structures and improved thin film crystallinities, in comparison with those prepared with spin‐coating, drop‐casting, and Langmuir‐Schaefer methods. Notably, thin film charge mobilities of conjugated polymers through **AOW** approach are boosted remarkably by comparing with the respective spin‐coated, drop‐casted, and Langmuir–Schaefer thin films. Moreover, this new approach can be utilized to easily fabricate the array of field‐effect transistors (FETs) with high charge mobilities in an economic way.

The **AOW** method is illustrated in **Figure** **1**. A solution of the polymer with low concentration (0.1 mg mL^−1^) was dropped onto the water, followed by transferring the assembly film to the substrate after evaporation of solvents (for details, see the Experimental Section). Thin films prepared with this facile approach were used for structural characterization and fabrication of FETs. We have employed this **AOW** approach to prepare thin films of six typical conjugated semiconducting polymers (see **Figures** **2** and **3**) in order to improve their thin film crystallinities and charge mobilities.

**Figure 1:**
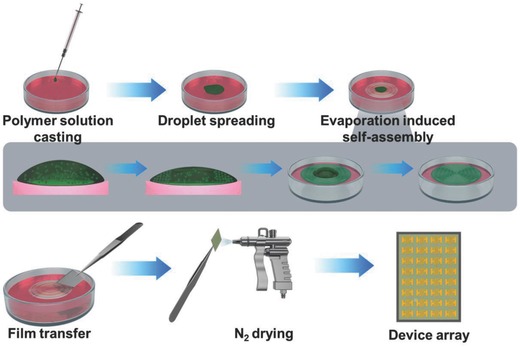
Schematic illustration of the thin films fabrication based on conjugated polymers by using the **AOW** method. Polymer solution is dropped on water surface to form a partly spread solution drop. As solvent evaporating at contact line, polymers gradually assemble at the edge and result in the formation of thin films with stripe‐like structures. Thin films can be transferred onto solid substrates and dried by blowing with dry N_2_, and the resulting thin films are used to fabricate field‐effect transistors.

**Figure 2:**
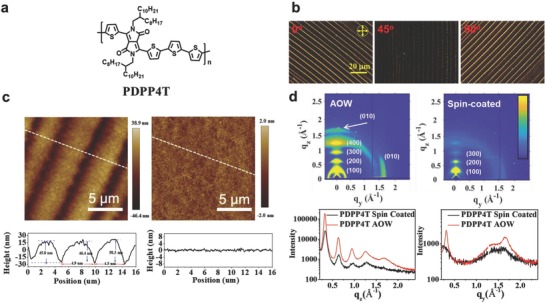
a) Chemical structure of **PDPP4T**; b) Polarized microscopy image of **PDPP4T** thin film; c) AFM height image of **PDPP4T** thin film prepared with the **AOW** method (left) and spin‐coated technique (right) with line‐cuts (below); d) GIWAXS 2D patterns of **PDPP4T** thin film prepared with the **AOW** method (left) and spin‐coated technique (right), and line‐cuts (below) at *q*_z_ (left) and *q*_y_ directions (right).

**Figure 3:**
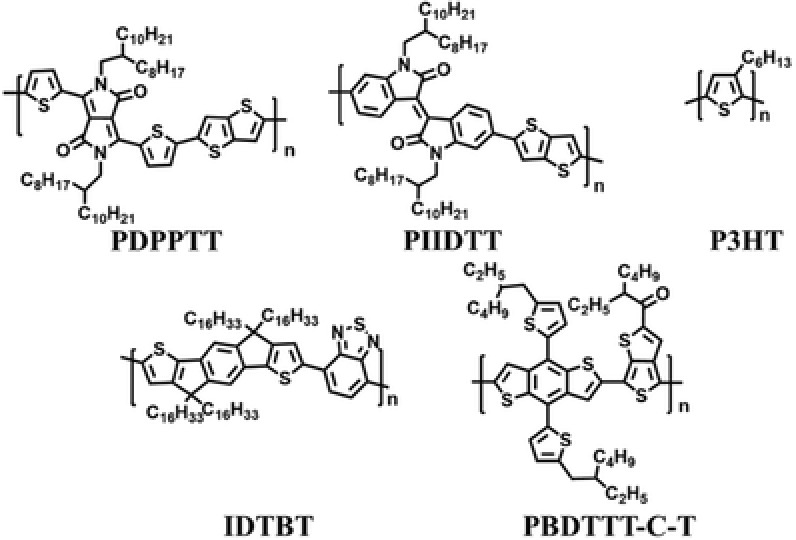
Chemical structures of semiconducting polymers.

First, we demonstrated the easiness and effectiveness of **AOW** approach with **PDPP4T** (Figure 2). 50 µL of **PDPP4T** solution was dropped on water surface at room temperature (25 °C). After about 3.0 min, thin film was formed (see Video S1 in Supporting Information) and easily transferred to solid substrates including glass, SiO_2_/Si, and OTS (octadecyltrichlorosilane)‐modified SiO_2_/Si for characterizations. The thin film obtained with this **AOW** approach was characterized with polarized optical microscopy (POM) and atomic force microscopy (AFM). As shown in Figure 2b, bright stripes from POM images were observed initially for the **AOW** thin film of **PDPP4T** under crosspole conditions. By rotating the sample for 45°, parts of stripes became dark, but scattering bright spots were still observable. Bright stripes were detected again by further rotating the sample for 45° as shown in Figure 2b. Such variation of POM images implies that the stripes within the **AOW** thin film of **PDPP4T** are composed of crystalline domains, but they are not completely aligned. Based on the AFM image, the **AOW** thin film of **PDPP4T** contains regularly arranged stripes and grooves (Figure 2c). The average width and height of each stripe are around 5 µm and 50 nm, respectively, while the average height of each groove is about 5 nm. Such hierarchically organized structure hints the ordered arrangement of polymer chains within thin film of **PDPP4T** through the self‐assembly on water. In comparison, such well‐arranged stripes were not detected from the AFM image (Figure 2c) for the corresponding spin‐coated thin film of **PDPP4T**.

Figure 2d shows the 2D grazing‐incidence wide‐angle X‐ray scattering (GIWAXS) pattern for thin film of **PDPP4T** prepared with the **AOW** method and that with spin‐coated method for comparison. Thin film of **PDPP4T** assembled on water shows scattering signals at *q*_z_ direction (*out‐of‐plane*) up to fourth order at *q*_z_ = 0.32, 0.63, 0.95, and 1.26 Å^−1^, due to the lamellar stacking of alkyl chains with a *d*‐spacing of 19.4 Å. An additional weak and broad signal appears at *q*_z_ = 1.69 Å^−1^, which is attributed to the interchain π–π stacking with a distance of 3.7 Å. At *q*_y_ direction (*in‐plane*) a strong scattering signal emerges at *q*_y_ = 1.66 Å^−1^, corresponding to a π–π stacking distance of 3.8 Å, which is shorter than that of (3.9 Å) for the spin‐coated thin film of **PDPP4T**. Additionally, a sharp scattering signal at *q*_y_ = 0.31 Å^−1^ appears for the **AOW** thin film at *q*_y_ direction, which is not observable for the spin‐coated thin film of **PDPP4T**. Moreover, the scattering signals of the thin film assembled on water become sharper. For instance, as shown in Table S1 in the Supporting Information, the full width at half maximum for the signal at *q*_z_ = 0.32 Å^−1^ of (100) is 0.037 Å^−1^ for the thin film assembled on water, which is smaller than that of the respective scattering signal (0.060 Å^−1^) for the spin‐coated thin film. On the basis of these GIWAXS data, it can be concluded that polymer chains of **PDPP4T** are more orderly packed and thin film crystallinity is significantly improved by using the **AOW** method.

Encouraged by this discovery, we extended this facile approach to other semiconducting polymers including conjugated D–A polymers such as poly[2,5‐(2‐octyldodecyl)‐3,6‐diketopyrrolopyrrole‐*alt*‐5,5‐(2,5‐di(thien‐2‐yl)thieno[3,2‐b]thiophene)] (**PDPPTT**), poly[*N,N*′‐(2‐octyldodecanyl)‐isoindigo‐*alt*‐thieno[3,2‐b]thiophene)] (**PIIDTT**),**IDTBT,** and poly[(4,8‐*bis*(5‐(2‐ethylhexyl)thio‐ phen‐2‐yl)‐benzo‐[1,2‐b:4,5‐b′]dithiophene‐2,6‐diyl)‐*alt*‐(2‐(2‐ethylhexanoyl)‐thieno[3,4‐b]thiophen‐4,6‐diyl)] (**PBDTTT‐C‐T**) as well as poly(3‐hexylthiophene‐2,5‐diyl) (**P3HT**) (Figure 3). Figures S1–S3 in the Supporting Information show AFM and GIWAXS patterns for thin films of **PDPPTT**, **PIIDTT,** and **P3HT**, which were fabricated with **AOW** method. As detailed in the Supporting Information, polymer chains of **PDPPTT**, **PIIDTT,** and **P3HT** are packed to form more ordered structures on substrates by using the **AOW** method. In comparison with the respective spin‐coated thin films, thin films of **PDPPTT**, **PIIDTT,** and **P3HT** prepared with the **AOW** method display improved crystallinity based on the GIWAXS data (Figures S1–S3 and Table S1, Supporting Information); Sharper scattering signals up to fourth and even fifth order (owing to the lamellar stacking of alkyl chains) and noticeable signals owing to the interchain π–π stacking were observed for these thin films with **AOW** method. For instance, both (300) and (400) scattering signals at *q*_z_ direction of the **AOW** thin film of **PDPPTT** become less dispersive than the respective ones of the spin‐coated thin film of **PDPPTT** on the basis of 2D GIWAXS patterns shown in Figure S1c in the Supporting Information. For **PIIDTT**, the (500) signal at *q*_z_ direction due to the lamellar stacking of alkyl chains was observed for the **AOW** thin film, whereas it was not detected for the spin‐coated one. Moreover, polymer chains of **PDPPTT**, **PIIDTT,** and **P3HT** are predominately arranged in the *edge‐on* mode on the substrate with the **AOW** method. In comparison, both *face‐on* and *edge‐on* packing modes coexist within the spin‐coated thin films of **PDPPTT** and **PIIDTT**, and polymeric chains of **P3HT** are packed dominantly with the *face‐on* mode for the spin‐coated thin film.

We also investigated conjugated polymers **PBDTTT‐C‐T** and **IDTBT** with the **AOW** approach. As shown in Figures S4 and S5 in the Supporting Information, thin films of **PBDTTT‐C‐T** and **IDTBT** show only broad and weak scattering signals and thus they are almost amorphous, being similar to the respective spin‐coated thin films. Therefore, this **AOW** approach cannot improve thin film crystallinities for **PBDTTT‐C‐T** and **IDTBT** whose spin‐coated thin films are also amorphous. This may be due to their low crystallinity natures as reported before.^15^, ^16^ These results demonstrate that this **AOW** approach is effective for conjugated polymers which exhibit tendency to form crystalline thin films, while this approach is not applicable for these conjugated polymers which are less inclined to assemble into ordered structures.

The formation of ordered structures through **AOW** method is attributed to “stick‐and‐slip”^20^ motion induced by solvent evaporation at contact line of solution droplet. As an example, the self‐assembly process of **PDPP4T** on water was investigated with optical microscopy. As shown in **Figure** **4**a and the Video S1 in Supporting Information, in which the pink and green areas represent water and polymer solute, respectively, the evaporation of solvents led to the repeated “stick‐and‐slip” motion of contact line. As a result, green stripes owing to the pinning of polymer assemblies were gradually formed. The formation of such green stripes is likely caused by coffee‐ring effect as schematically illustrated in Figure 4b. Moreover, the hydrophobic nature of these polymers forces the polymer chains to pack densely with *edge‐on* mode in order to minimize the contact of polymer chains with water. This agrees well with the observation that polymer chains of **PDPP4T**, **PDPPTT**, **PIIDTT,** and **P3HT** adopt predominant *edge‐on* mode on the basis of 2D GIWAXS data.

**Figure 4:**
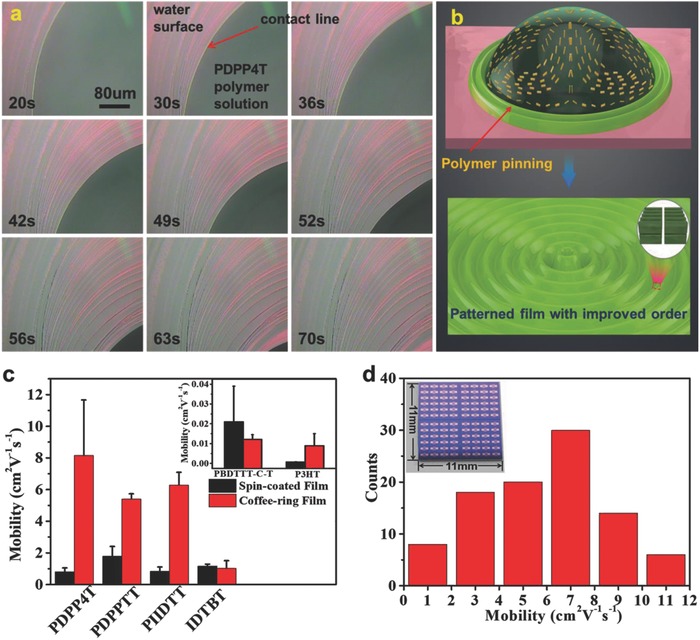
a) Microscopic observation of **PDPP4T** during the formation process of stripes on water surface. The pink areas represent water surfaces, while the dark‐green areas represent polymer (**PDPP4T**) solution droplets, and pale green annular stripes of polymer assemblies are formed on pink region. b) Schematic illustration of coffee ring effect induced assembly of polymer on water. c) Hole mobilities of thin films of investigated polymers prepared with **AOW** and spin‐coated approaches. All data were based on statistics of at least 50 BGBC FET devices with *W* = 1400 µm, *L* = 50 µm. The mobilities of **PDPP4T**, **PDPPTT**, and **PIIDTT** were extracted at low *V*_G_ region. d) Hole mobility distribution for 8 × 12 FETs array fabricated with **AOW** method.

The solvent evaporation process affects the formation of ordered structures for conjugated polymers with **AOW** method. Among the solvents which can dissolve **PDPP4T**, chloroform is the best solvent for the formation of ordered **AOW** thin film. For example, thin film with stripe‐like structures was not formed on the basis of the AFM image (see Figure S6, Supporting Information) when the polymer solution was prepared with chlorobenzene which owns lower volatility than chloroform. We also prepared **AOW** thin films of **PDPP4T** at 0 and 31 °C (besides 25 °C) by using chloroform as the solvent. In both cases thin films with regularly arranged stripes were obtained as shown in Figure S7 in the Supporting Information in which AFM images of these two thin films are displayed. This indicates the effect of temperature on the formation of **AOW** thin films with ordered structures is weak in the temperature range of 0–31 °C because of the relatively high volatility of chloroform.

It was reported that such “stick‐and‐slip” motion of contact line led to the assembly of nonconjugated polymers into ordered structures on solid substrate (not on water surface).^20^, ^21^, ^22^ Notably, the solvent‐evaporation induced self‐assembly with **AOW** approach occurs on water surface, being different from previous reports in which the assembly of organic and polymeric semiconductors into ordered structures was conducted on solid substrates in confined geometries.^23^, ^24^, ^25^ The assembly of a few small conjugated molecules on water to form crystalline thin films with high charge mobilities has been recently reported.^26^, ^27^ In fact, we employed spin‐coated and drop‐casted methods to prepare thin films of **PDPP4T** on plasma treated SiO_2_/Si substrate which shows lower contact angle (35.7°) and thus higher surface energy than OTS‐modified SiO_2_/Si substrate with a contact angle of 83.8° (Figure S8a, Supporting Information). Figure S8b in the Supporting Information displays the AFM images of these thin films. Clearly, these thin films exhibit no stripe‐like structures. This result manifests that self‐assembly of conjugated polymers on water surface is important to form semiconducting thin films with ordered structures by using **AOW** method.

The **AOW** thin film of **PDPP4T** was also transferred onto plasma treated SiO_2_/Si substrate which shows higher surface energy than OTS‐modified SiO_2_/Si substrate. As shown in Figure S8 in the Supporting Information, stripe‐like structure was retained and was not obviously influenced by the surface energy feature of substrates. Moreover, the possibility of further assembly of polymer chains on the substrate owing to water evaporation can be ruled out because morphologies of thin films of **PDPP4T** were almost the same although they were prepared in different humidities ranging from 28% to 90% relative humidity (RH).

Based on the fact that thin films of **PDPP4T**, **PDPPTT**, **PIIDTT,** and **P3HT** prepared with the **AOW** approach show improved crystallinities as discussed above, these thin films are expected to exhibit high charge mobilities. Thin film charge mobilities were extracted on the basis of the semiconducting performances of the respective bottom gate/bottom contact (BGBC) FETs. As an example, Figure S11a,A in the Supporting Information shows the transfer and output curves of the FETs with thin films of **PDPP4T** through **AOW** approach and spin‐coated method, respectively. Obviously, the on‐state current (*I*_on_, ≈7.68 × 10^−4^ A at *V*_GS_ = −40 V, *V*_DS_ = −100 V) for the FET with the **AOW** thin film is much higher than that (≈1.14 × 10^−4^ A) for the spin‐coated thin film. We note that the transfer characteristics are not ideal, which is probably due to contact issues in BGBC FET devices. In fact, such nonideal transfer characteristics was observed for FETs with conjugated D–A polymers.^28^ The hole charge mobilities were extracted by fitting the respective plots of *I*_DS_^1/2^ versus *V*_G_ at both low *V*_G_ and high *V*_G_ regions as shown in Figure S11 in the Supporting Information.^28^ As listed in **Table** **1**, the average (μ^s^) and maxima saturated mobilities (μ_max_^s^) are 8.15 and 11.66 cm^2^ V^−1^ s^−1^ at low *V*_G_ region, respectively, for the as‐prepared **AOW** thin film of **PDPP4T**. In comparison, the respective μ^s^ and μ_max_^s^ are just 0.8 and 1.05 cm^2^ V^−1^ s^−1^ for the spin‐coated thin film of **PDPP4T** without thermal treatment.^29^ The average charge mobilities by fitting at high *V*_G_ region are 2.01 and 0.22 cm^2^ V^−1^ s^−1^ for the **AOW** and the spin‐coated thin films of **PDPP4T**, respectively (see μ'^s^ in Table 1). Moreover, the **AOW** thin film of **PDPP4T** also exhibits higher linear mobility (2.01 cm^2^ V^−1^ s^−1^) than the spin‐coated thin film (0.27 cm^2^ V^−1^ s^−1^) as listed in Table 1. These data clearly indicate that thin film charge mobility can be dramatically boosted for **PDPP4T** by employing the **AOW** method without additional post‐treatments such as thermal annealing (Figure 4c). We also compared the FET mobilities of **PDPP4T** thin films fabricated by using **AOW** and spin‐coated methods after thermal annealing. As shown in Table S2 in the Supporting Information, the average mobility (by fitting at low *V*_G_ region) of the **AOW** thin film of **PDPP4T** was boosted to 9.97 cm^2^ V^−1^ s^−1^ after thermal annealing at 120 °C. Under the same annealing condition, the average mobility of the spin‐coated thin film of **PDPP4T** was enhanced only to 2.94 cm^2^ V^−1^ s^−1^. Additionally, it is noted that the *on–off* current ratio (*I*_on_/*I*_off_), threshold voltage (*V*_Th_) for the **AOW** thin film of **PDPP4T** are comparable to those of the spin‐coated thin film (see Table 1).

**Table 1:** Hole mobilities (saturated mobilities, maxima saturated mobilities, linear mobilities, and maxima linear mobilities), On currents, *I*_on_/*I*_off_ ratios, threshold voltages, subthreshold slopes of BGBC FETs with thin films of **PDPP4T**, **PDPPTT**, **PIIDTT**, **P3HT**, **PBDTTT‐C‐T**, and **IDTBT** prepared with **AOW**, and spin‐coated approaches

Polymer	Processing method	μ^s^ (μ_m_ *_ax_* ^s^)a) [cm^2^ V^−1^ s^−1^]	μ'^s^ (μ'_max_^s^)b) [cm^2^ V^−1^ s^−1^]	μ^lin^ [cm^2^ V^−1^ s^−1^]	μ_m_ *_ax_* ^lin^ [cm^2^ V^−1^ s^−1^]	*I*_on_ [A]	*I*_on_/*I*_off_ [log 10]	*V*_Th_ [V]	Subthreshold Slopes [V dec^−1^]
**PDPP4T**	Spin‐coating	0.80 ± 0.16 (1.05)	0.22 ± 0.01 (0.24)	0.27 ± 0.05	0.33	1.14 × 10^−4^	6–7	1 to 7	2.6–2.8
	**AOW**	8.15 ± 2.52 (11.66)	2.01 ± 0.25 (2.32)	2.01 ± 0.63	2.71	7.68 × 10^−4^	6–7	−1 to 4	1.1–1.3
**PDPPTT**	Spin‐coating	1.79 ± 0.54 (2.41)	0.18 ± 0.02 (0.22)	0.31 ± 0.09	0.45	1.05 × 10^−4^	6–7	−3 to 2	2.3–2.4
	**AOW**	5.40 ± 0.30 (5.73)	1.59 ± 0.17 (1.78)	1.03 ± 0.08	1.15	9.05 × 10^−4^	5–6	0 to 6	1.9–2.1
**PIIDTT**	Spin‐coating	0.84 ± 0.24 (1.11)	0.11 ± 0.03 (0.15)	0.17 ± 0.03	0.21	4.81 × 10^−5^	7–8	1 to 5	2.0–2.3
	**AOW**	6.28 ± 0.69 (7.09)	2.30 ± 0.22 (2.54)	1.52 ± 0.22	1.97	1.03 × 10^−3^	7–8	−3 to 3	1.1–1.2
**P3HT**	Spin‐coating	7.3 × 10^−4^ ± 0.2 × 10^−4^ (7.6 × 10^−4^)		2.9 × 10^−4^ ± 0.2 × 10^−4^	3.2 × 10^−4^	1.58 × 10^−7^	3–4	0 to 5	8.1–9.1
	**AOW**	8.9 × 10^−3^ ± 1.8 × 10^−3^ (1.5 × 10^−2^)		4.1 × 10^−4^ ± 1.9 × 10^−4^	4.2 × 10^−3^	1.45 × 10^−6^	3–4	−3 to 2	7.5–8.4
**IDTBT**	Spin‐coating	1.16 ± 0.09 (1.29)		0.60 ± 0.02	0.64	2.07 × 10^−4^	6–7	0 to 4	1.1–1.3
	**AOW**	1.02 ± 0.36 (1.51)		0.54 ± 0.18	0.56	2.08 × 10^−4^	7–8	−6 to −1	1.5–1.7
**PBDTTT‐C‐T**	Spin‐coating	0.021 ± 0.012 (0.039)		0.013 ± 0.002	0.016	8.43 × 10^−6^	5–6	−1 to 3	1.0–1.3
	**AOW**	0.012 ± 0.001 (0.015)		0.0089 ± 0.0002	0.0092	2.19 × 10^−6^	5–6	1 to 5	2.3–2.5

^a)^ Saturated mobilities were extracted at low *V*_G_ region for polymer **PDPP4T**, **PDPPTT**, and **PIIDTT**

^b)^ Saturated mobilities were extracted at high *V*_G_ region for polymer **PDPP4T**, **PDPPTT**, and **PIIDTT**, whose transfer characteristics are not ideal. When averaging charge mobility, data were based on statistics of at least 50 BGBC FET devices with *W* = 1400 µm and *L* = 50 µm.

The following control experiments by using drop‐casting and Langmuir–Schaefer methods further demonstrate the advantage of **AOW** approach. We dropped the solution of **PDPP4T** with a concentration of 0.1 mg mL^−1^ onto the OTS/SiO_2_/Si substrate, and the resulting thin film after drying shows poor charge‐transporting performance with the maxima and average mobilities of 0.24 and 0.15 cm^2^ V^−1^ s^−1^,^30^ which were measured under the same conditions as for the thin film of **PDPP4T** fabricated with **AOW** approach. This agrees well with the observation that the drop‐casting thin film shows no stripe‐like structure (see the AFM image in Figure S9a, Supporting Information). In fact, this thin film contains random aggregates of different sizes. This is also confirmed by the GIWAXS result (Figure S9b, Supporting Information), which showed low crystallinity compared to that by using **AOW** method. Thus, the evaporation‐driven self‐assembly of polymer chains *on water* is vital for the formation of ordered structure with high crystallinity for **PDPP4T**. Alternatively, thin films of **PDPP4T** were also prepared with Langmuir–Schaefer technique at air–water interface at different surface pressures according to the surface pressure (π) versus mean monomeric area isotherm (Figure S10a, Supporting Information). The resulting thin films show poor morphology and low crystallinity based on their AFM images and GIWAXS results (see Figure S10b,c, Supporting Information) with low charge mobilities. For instance, the average hole mobility for the thin film prepared at 40 mN m^−1^ was measured to 0.003/0.0049 cm^2^ V^−1^ s^−1^, being much lower than that of the **AOW** thin film.

Such mobility enhancement also holds true for **PDPPTT**, **PIIDTT**, and **P3HT** by using the **AOW** approach (see Figure 4c and Figure S11, Supporting Information, and Table 1). The transfer and output curves of FETs with both **AOW** and spin‐coated thin films of **PDPPTT**, **PIIDTT**, and **P3HT** are shown in Figure S11 in the Supporting Information. For **PDPPTT**, the average and maxima saturated mobilities are incremented from 1.79 and 2.41 cm^2^ V^−1^ s^−1^ for the spin‐coated thin film to 5.40 and 5.73 cm^2^ V^−1^ s^−1^ (extracted at low *V*_G_ region), respectively, with the **AOW** approach. The linear charge mobility (μ^lin^) also increases from 0.31 cm^2^ V^−1^ s^−1^ to 1.03 cm^2^ V^−1^ s^−1^ by employing the **AOW** approach (see Table 1). Both saturated and linear mobilities are enhanced by approximately three times for **PDPPTT** by using the **AOW** approach. Similarly, charge mobilities can reach 7.09 and 1.5 × 10^−2^ cm^2^ V^−1^ s^−1^, being approximately seven and ten times of the respective spin‐coated thin films, for **PIIDTT** and **P3HT** by using the **AOW** approach, whereas the *on–off* current ratio (*I*_on_/*I*_off_), threshold voltage (*V*_Th_) are not affected (see Table 1). As reported early, the transfer characteristics of FETs with thin films of **PDPPTT** and **PIIDTT** is nonideal (see Figures S11, Supporting Information). Their charge mobilities were extracted by fitting the respective plots of *I*_DS_^1/2^ versus *V*_G_ at both low *V*_G_ and high *V*_G_ regions. As listed in Table 1, the average charge mobilities (extracted at high *V*_G_ region) of **AOW** thin films of **PDPPTT** and **PIIDTT** are higher than those of the respective spin‐coated thin films.

For **IDTBT**, both the saturated (1.02 cm^2^ V^−1^ s^−1^) and linear (0.54 cm^2^ V^−1^ s^−1^) charge mobilities for the **AOW** thin film are comparable to those of the spin‐coated thin film, while charge mobilities for **PBDTTT‐C‐T** are reduced by employing the **AOW** approach. This is indeed in agreement with the fact that thin film crystallinity is not improved for **IDTBT** and **PBDTTT‐C‐T** by using **AOW** approach as discussed above.

This facile **AOW** approach can be utilized to easily fabricate the array of FETs in an economic way. As an example, 50 µL (0.1 mg mL^−1^ in chloroform) of **PDPP4T** was dropped onto the water surface and the resulting thin film was transferred onto the OTS‐modified Si/SiO_2_ substrate on which the 8 × 12 array of source/drain Au electrodes were deposited. As a result, the 8 × 12 array of BGBC FETs was fabricated. Figure 4d shows the distribution of hole mobilities of the 96 FETs. The average mobility is 6.78 cm^2^ V^−1^ s^−1^ (extracted at low *V*_G_ region),^31^ and 81% of the devices exhibit mobilities higher than 3.0 cm^2^ V^−1^ s^−1^. It is noted that ≈20 µL of the polymer solution (5.0 mg mL^−1^) is needed usually to fabricate 8 × 12 array of FETs with the spin‐coated technique. Therefore, this **AOW** approach is not only simple and facile but also economic for the fabrication of arrays of FETs, which are potentially useful for organic circuits.

In addition, FETs with **AOW** approach show relatively good operational stabilities. As shown in Figure S12a in the Supporting Information, the *on* and *off* currents of the FET with the assembly‐on‐water thin film of **PDPP4T** kept nearly constant by switching gate voltage between 0 and −30 V for over 700 cycles. We also did the double‐sweep measurements for the transfer and output curves of FETs with **AOW** thin films of six polymers. As shown in Figure S13 in the Supporting Information, both transfer and output curves exhibit rather small hysteresis. The possible water residues in the **AOW** thin films, which may affect the device stability, can be easily removed by either blowing the thin films with dry N_2_ or treating them under vacuum. Moreover, the semiconducting performance of FETs fabricated with this **AOW** approach is weakly affected by environmental humidity. As an example, the charge mobility for thin film of **PDPP4T** prepared in air with 90% humidity is slightly reduced by comparing with that for the same **AOW** thin film prepared in air with 20% humidity as shown in Figure S12b in the Supporting Information. This may be attributed to the fact that **AOW** thin films show improved crystallinity, which (plus the hydrophobic nature of these polymers) can prevent the penetration of water molecules into the thin films.

We have developed a facile and economic approach to assemble conjugated polymers into thin films with improved crystallinities. This is referred to as **AOW** approach. Six typical semiconducting polymers **PDPP4T**, **PDPPTT**, **PIIDTT**, **P3HT**, **IDTBT,** and **PBDTTT‐C‐T** were investigated. On the basis of GIWAXS and AFM data, polymeric chains of **PDPP4T**, **PDPPTT**, **PIIDTT,** and **P3HT** are assembled into more ordered structures with this **AOW** approach. Interestingly, polymer chains within thin films of these polymers adopt predominantly *edge‐on* packing mode on substrates. The formation of more ordered structures is induced by the “stick‐and‐slip” motion of contact line after solvent evaporation and the hydrophobic nature of these polymers, which forces the polymer chains to pack densely with predominant *edge‐on* mode in order to minimize the contact of polymer chains with water. Further studies show that this method is particularly effective for conjugated polymers which tend to form crystalline thin films. In comparison with those of spin‐coated, drop‐casted, and Langmuir‐Schaefer thin films, charge mobilities for thin films of **PDPP4T**, **PDPPTT**, **PIIDTT,** and **P3HT** are boosted significantly by using **AOW** approach. Moreover, the method is simple and effective to fabricate FET arrays with high average mobility of 6 cm^2^ V^−1^ s^−1^. Therefore, this facile and economic approach for assembling semiconducting polymers into more ordered structures with high charge mobilities is potentially useful for the fabrication of organic circuits.

## Experimental Section

*Fabrication of Polymer Thin Films by Using* ***AOW*** *Method*: All conjugated polymers were dissolved in chloroform with a concentration of 0.1 mg mL^−1^. Specifically, the chloroform solutions of **PDPP4T**, **P3HT**, **PBDTTT‐C‐T,** and **IDTBT** were prepared by stirring the solutions overnight at ambient condition, while those of **PDPPTT** and **PIIDTT** were formed by heating at 50 °C under nitrogen for 12 h. For film growth on water surface, 40 mL of deionized water was added into a vessel (diameter 60 mm, height 30 mm), and 50 µL of the polymer solution (0.1 mg mL^−1^) was dropped onto the water surface with microsyringe. The growth of film was completed during several minutes after evaporation of solvents. Then, a substrate (glass, SiO_2_/Si or OTS‐modified SiO_2_/Si with gold electrodes) was inserted at an angle into the water to transfer thin films on air–water interface to the substrate, and thin film was blown with dry nitrogen to remove residual water. The average thicknesses of stripes/grooves (area between stripes) of **AOW** thin films were 50 nm/5 nm for **PDPP4T**, 22 nm/8 nm for **PDPPTT**, 40 nm/17 nm for **PIIDTT**, 65 nm/5 nm for **P3HT**, 29 nm/4 nm for **IDTBT**, and 33 nm/10 nm for **PBDTTT‐C‐T**. The characterizations of **AOW** thin films with POM, GIWAXS, and AFM, fabrication of FETs with **AOW** thin films, and the comparative experiments are included in the Supporting Information.

## Conflict of Interest

The authors declare no conflict of interest.

## Supporting information

Supplementary

Supplementary

## References

[1] H. Sirringhaus , Adv. Mater. 2014, 26, 1319. 24443057 10.1002/adma.201304346 PMC4515091

[2] Y. Yuan , G. Giri , A. L. Ayzner , A. P. Zoombelt , S. C. B. Mannsfeld , J. Chen , D. Nordlund , M. F. Toney , J. Huang , Z. Bao , Nat. Commun. 2014, 5, 3005. 24398476 10.1038/ncomms4005

[3] C. Luo , A. K. K. Kyaw , L. A. Perez , S. Patel , M. Wang , B. Grimm , G. C. Bazan , E. J. Kramer , A. J. Heeger , Nano Lett. 2014, 14, 2764. 24712578 10.1021/nl500758w

[4] H. Luo , C. Yu , Z. Liu , G. Zhang , H. Geng , Y. Yi , K. Broch , Y. Hu , A. Sadhanala , L. Jiang , P. Qi , Z. Cai , H. Sirringhaus , D. Zhang , Sci. Adv. 2016, 2, e1600076. 27386541 10.1126/sciadv.1600076 PMC4928946

[5] Y. Wen , Y. Liu , Y. Guo , G. Yu , W. Hu , Chem. Rev. 2011, 111, 3358. 21401072 10.1021/cr1001904

[6] C. B. Nielsen , M. Turbiez , I. McCulloch , Adv. Mater. 2013, 25, 1859. 23008141 10.1002/adma.201201795

[7] Y. Zhao , Y. Guo , Y. Liu , Adv. Mater. 2013, 25, 5372. 24038388 10.1002/adma.201302315

[8] T. Lei , J.‐Y. Wang , J. Pei , Acc. Chem. Res. 2014, 47, 1117. 24502431 10.1021/ar400254j

[9] H. N. Tsao , K. Müllen , Chem. Soc. Rev. 2010, 39, 2372. 20386811 10.1039/b918151m

[10] W. Li , K. H. Hendriks , M. M. Wienk , R. A. J. Janssen , Acc. Chem. Res. 2016, 49, 78. 26693798 10.1021/acs.accounts.5b00334

[11] J. Y. Oh , S. Rondeau‐Gagné , Y.‐C. Chiu , A. Chortos , F. Lissel , G.‐J. N. Wang , B. C. Schroeder , T. Kurosawa , J. Lopez , T. Katsumata , J. Xu , C. Zhu , X. Gu , W.‐G. Bae , Y. Kim , L. Jin , J. W. Chung , J. B.‐H. Tok , Z. Bao , Nature 2016, 539, 411. 27853213 10.1038/nature20102

[12] V. Coropceanu , J. Cornil , D. A. d. S. Filho , Y. Olivier , R. Silbey , J.‐L. Brédas , Chem. Rev. 2007, 107, 926. 17378615 10.1021/cr050140x

[13] Z. Shuai , H. Geng , X. Wei , Y. Liao , J.‐M. André , Chem. Soc. Rev. 2014, 43, 2662. 24394992 10.1039/c3cs60319a

[14] S. Himmelberger , A. Salleo , MRS Commun. 2015, 5, 383.

[15] R. Noriega , J. Rivnay , K. Vandewal , F. P. V. Koch , N. Stingelin , P. Smith , M. F. Toney , A. Salleo , Nat. Mater. 2013, 12, 1038. 23913173 10.1038/nmat3722

[16] X. Zhang , H. Bronstein , A. J. Kronemeijer , J. Smith , Y. Kim , R. J. Kline , L. J. Richter , T. D. Anthopoulos , H. Sirringhaus , K. Ong , M. Heeney , W. Zhang , I. McCulloch , D. M. Delongchamp , Nat. Commun. 2013, 4, 2238. 23900027 10.1038/ncomms3238

[17] M. J. Lee , D. Gupta , N. Zhao , M. Heeney , I. McCulloch , H. Sirringhaus , Adv. Funct. Mater. 2011, 21, 932.

[18] J. Lee , A. R. Han , H. Yu , T. J. Shin , C. Yang , J. H. Oh , J. Am. Chem. Soc. 2013, 135, 9540. 23711152 10.1021/ja403949g

[19] H.‐R. Tseng , H. Phan , C. Luo , M. Wang , L. A. Perez , S. N. Patel , L. Ying , E. J. Kramer , T.‐Q. Nguyen , G. C. Bazan , A. J. Heeger , Adv. Mater. 2014, 26, 2993. 24504475 10.1002/adma.201305084

[20] W. Sun , F. Yang , Langmuir 2015, 31, 4024. 25785552 10.1021/acs.langmuir.5b00230

[21] W. Bi , X. Wu , E. K. L. Yeow , Langmuir 2012, 28, 11056. 22747256 10.1021/la300697w

[22] W. Sun , F. Yang , Soft Matter 2014, 10, 4451. 24803223 10.1039/c4sm00245h

[23] W. Han , M. He , M. Byun , B. Li , Z. Lin , Angew. Chem., Int. Ed. 2013, 52, 2564. 10.1002/anie.201209632 23355504

[24] L. Jiang , H. Dong , Q. Meng , H. Li , M. He , Z. Wei , Y. He , W. Hu , Adv. Mater. 2011, 23, 2059. 21381133 10.1002/adma.201004551

[25] Q. Wang , J. Qian , Y. Li , Y. Zhang , D. He , S. Jiang , Y. Wang , X. Wang , L. Pan , J. Wang , X. Wang , Z. Hu , H. Nan , Z. Ni , Y. Zheng , Y. Shi , Adv. Funct. Mater. 2016, 26, 3191.

[26] C. Xu , P. He , J. Liu , A. Cui , H. Dong , Y. Zhen , W. Chen , W. Hu , Angew. Chem., Int. Ed. 2016, 55, 9519. 10.1002/anie.201602781 27237452

[27] Q. Wang , F. Yang , Y. Zhang , M. Chen , X. Zhang , S. Lei , R. Li , W. Hu , J. Am. Chem. Soc. 2018, 140, 5339. 29522681 10.1021/jacs.8b01997

[28] I. McCulloch , A. Salleo , M. Chabinyc , Science 2016, 352, 1521. 27339971 10.1126/science.aaf9062

[29] It should be noted that the thin film mobilities fabricated by **AOW** method are higher than those of spin‐coated ones, although the thin **AOW** films are not even in thickness. The good tolerance of thin film thickness by using **AOW** method is beneficial for future large‐area device fabrications.

[30] Thin film of **PDPP4T** was also prepared on plasma treated SiO_2_/Si substrate with drop‐casting method. The thin film shows no stripe‐like structure on the basis of the AFM image (see Figure S8b, Supporting Information). This thin film exhibits even poor semiconducting performance with average charge mobility of 0.0066 cm^2^ V^−1^ s^−1^ because of the presence of –OH groups (as charge carrier traps) at the interface. Similarly, the spin‐coated thin film of **PDPP4T** on plasma treated SiO_2_/Si substrate also shows poor morphology and semiconducting performance with average charge mobility of 0.0073 cm^2^ V^−1^ s^−1^.

[31] The average charge mobility extracted at high *V* _G_ region can reach 1.71 cm^2^ V^−1^ s^−1^.

