# Supplementary material for: A Facile Approach to Improve Interchain Packing Order and Charge Mobilities by Self‐Assembly of Conjugated Polymers on Water
Source: Adv Sci (Weinh). 2018 Oct 9;5(11):1801497. doi: 10.1002/advs.201801497 (PMC6247062; doi:10.1002/advs.201801497)
Supplement: Supplementary file 1 — Supplementary [file ADVS-5-1801497-s002.pdf]

Copyright WILEY-VCH Verlag GmbH & Co. KGaA, 69469 Weinheim, Germany, 2018.

ADVANCED SCIENCE
Open Access

Supporting Information

for *Adv. Sci.,* DOI: 10.1002/advs.201801497

A Facile Approach to Improve Interchain Packing Order and Charge Mobilities by Self-Assembly of Conjugated Polymers on Water

Yizhou Yang, Zitong Liu, Jianmei Chen, Zhengxu Cai, Zhijie Wang, Wei Chen, Guanxin Zhang, Xisha Zhang, Lifeng Chi,* and Deqing Zhang**

Copyright WILEY-VCH Verlag GmbH & Co. KGaA, 69469 Weinheim, Germany, 2018.

Supporting Information

A facile approach to improve interchain packing order and charge mobilities by self-assembly of conjugated polymers on water

Yizhou Yang, Zitong Liu, Jianmei Chen, Zhengxu Cai, Zhijie Wang, Wei Chen, Guanxin Zhang, Xisha Zhang, Lifeng Chi*, Deqing Zhang**

Y. Yang, Dr. Z. Liu, Dr. Z. Wang, Prof. G. Zhang, Dr. X. Zhang, Prof. D. Zhang
Beijing National Laboratory for Molecular Sciences, CAS Key Laboratory of Organic Solids, CAS Center of Excellence in Molecular Science, Institute of Chemistry, Chinese Academy of Sciences, Beijing, 100190, P. R. China
E-mail: dqzhang@iccas.ac.cn; zitong_@iccas.ac.cn

Y. Yang, Dr. Z. Wang, Dr. X. Zhang, Prof. D. Zhang
School of Chemical Sciences, University of Chinese Academy of Sciences, Beijing 100049, P. R. China

Dr. J. Chen, Prof. L. Chi
Institute of Functional Nano and Soft Materials (FUNSOM), Jiangsu Key Laboratory for Carbon-Based Functional Materials and Devices, Soochow University, Suzhou 215123, China
E-mail: chilf@suda.edu.cn

Dr. Z. Cai
Beijing Key Laboratory of Construction Tailorable Advanced Functional Materials and Green Applications, School of Material Science & Engineering, Beijing Institute of Technology, Beijing 100081, China

Dr. W. Chen
Institute for Molecular Engineering and Materials Science Division, Argonne National Laboratory, 9700 Cass Avenue, Lemont, Illinois, 60439, United States

Dr. W. Chen
Institute for Molecular Engineering, The University of Chicago, 5640 South Ellis Avenue, Chicago, Illinois, 60637, United States

1. Materials and Characterization techniques 3

2. Characterizations of thin films of other polymers by using **AOW** and spin-coating methods 4-9

3. Characterizations of **PDPP4T** thin films by using **AOW** method and other methods under different conditions 10-14

4. Fabrications and characterizations of FET devices and corresponding semiconducting data 15-20

5. Reference 20

1. Materials and Characterization techniques

Materials: Polymers **PDPP4T** (M_w = 186 kDa, *Đ* = 2.6), **PDPPTT** (M_w = 271 kDa, *Đ* = 2.9), **PIIDTT** (M_w = 257 kDa, *Đ* = 3.0) were prepared and purified according to the reported procedures.[S1-S2] **P3HT** (M_w = 50 kDa, *Đ* = 2.0), **IDTBT** (M_w = 132 kDa, *Đ* = 2.7), **PBDTTT-C-T** (M_w = 18 kDa, *Đ* = 1.8) were purchased from Solarmer Materials Inc. and used as received. All solvents were purchased from Sigma-Aldrich and Acros Organics and used directly.

Characterization techniques: The photos and videos were taken by SONY SSC-G803. Polarized optical images were taken on OLYMPUS BX51 by using halogen lamp. Atomic-force microscopy images of thin-films of polymer were taken by using an Asylum Research Cypher S atomic force microscope operated in tapping mode with a Cypher Sinstrument in air. The film thickness was measured with DektakXT Bruker Stylus Profiler. The GIWAXS measurements were performed at the 8ID-E beamline at the Advanced Photon Source (APS), Argonne National Laboratory, using X-rays with a wavelength of λ = 1.136 Å and a beam size of ~200 μm (h) and 20 μm (v). Typical GIWAXS patterns were taken at an incidence angle of 0.13°, which was above the critical angles of neat polymers and below the critical angle of silican wafer substrate. Consequently, the entire structure of thin films can be detected. In addition, the q_y linecut was obtained from a linecut across the reflection beam center, while the q_z linecut was achieved by a linecut at $q_y = 0$ Å^{-1}.

2. Characterizations of thin films of other polymers by using AOW and spin-coating methods

2.1 Chemical structure, AFM images and GIWAXS patterns of PDPPTT thin film

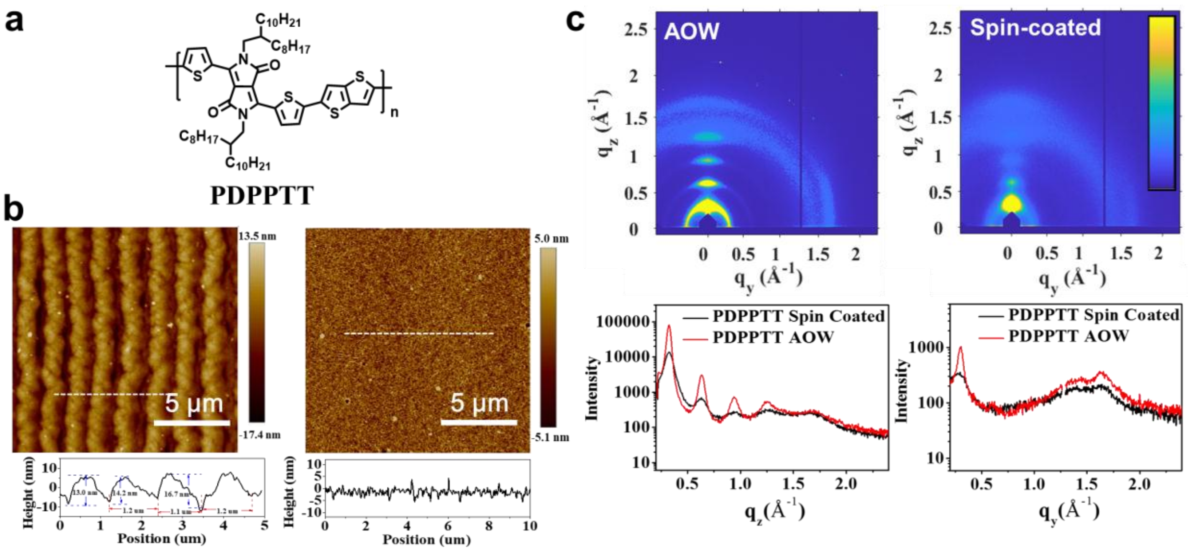

Figure S1. Chemical structure, AFM images and GIWAXS patterns of **PDPPTT** thin film. **a**, Chemical structure of **PDPPTT**; **b**, AFM height image of **PDPPTT** thin film with **AOW** method (*left*) and spin-coated film (*right*) with line-cuts (*below*); **c**, 2D GIWAXS patterns of **PDPPTT** thin film prepared with the **AOW** method (*left*) and spin-coated technique (*right*), and line-cuts (*below*) at q_z (*left*) and q_y directions (*right*).

Thin film of **PDPPTT** is composed of regularly-arranged stripes, and the width and maxima height of each stripe are around 1.2 μm and 16 nm, respectively. AFM image of **PDPPTT** shown in Figure S1 is obviously different from that of the respective spin-coated thin films. Importantly, thin film of **PDPPTT** fabricated with the **AOW** approach displays improved crystallinity comparing with the respective spin-coated thin film on the basis of 2D GIWAXS patterns shown in Figure S1. For thin film of **PDPPTT**, scattering signals up to fourth order owing to lamellar stacking of side chains appear at q_z = 0.32, 0.63, 0.94, 1.25 Å^{-1}, corresponding to *d*-spacing of 19.4 Å. Particularly, all (X00) signals become sharper and the FWHMs (Table S1) are reduced by half in comparison with that of the spin-

coated thin film (see Table S1 below). For instance, the FWHM for the signal at q_z = 0.32 Å^{-1} of (100) is 0.036 Å^{-1} for the thin film assembled on water, which is smaller than that of the respective scattering (0.075 Å^{-1}) for the spin-coated thin film.

2.2 Chemical structure, AFM images and GIWAXS patterns of PIIDTT thin film

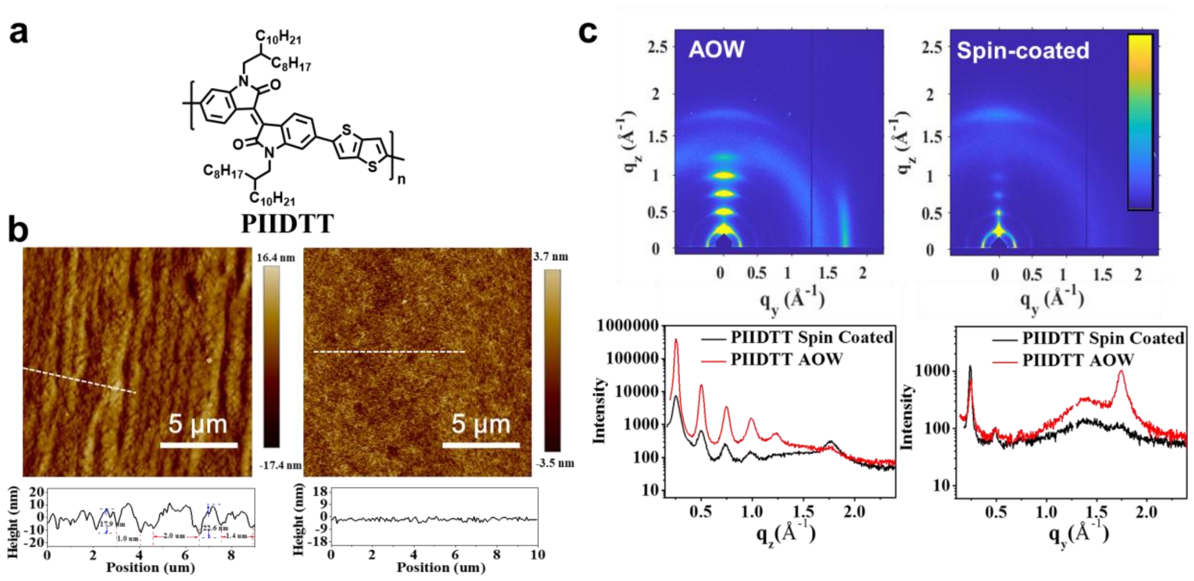

Figure S2. Chemical structure, AFM images and GIWAXS patterns of **PIIDTT** thin film. **a**, Chemical structure of **PIIDTT**; **b**, AFM height image of **PIIDTT** thin film with **AOW** method (*left*) and spin-coated film (right) with line-cuts (*below*); **c**, 2D GIWAXS patterns of **PIIDTT** thin film prepared with the **AOW** method (*left*) and spin-coated technique (*right*), and line-cuts (*below*) at q_z (*left*) and q_y directions (*right*).

AFM image of **PIIDTT** shown in Figure S2 is obviously different from that of the respective spin-coated thin film. Importantly, thin film of **PIIDTT** fabricated with the **AOW** approach displays improved crystallinity comparing with the respective spin-coated thin film on the basis of 2D GIWAXS patterns shown in Figure S2. Strong lamellar stacking signals up to fifth order were detected for thin film of **PIIDTT**, while only four lamellar signals with relatively weak intensities at q_z direction

emerged for the spin-coated thin film. Moreover, thin film of **PIIDTT** with the **AOW** approach shows a noticeable scattering at $q_y = 1.75$ Å^{-1}, corresponding to the interchain π-π stacking with a distance of 3.6 Å, while the spin-coated thin film displays broad scattering owing to the interchain packing at $q_z = 1.74$ Å^{-1}. Moreover, FWHMs of scattering singals for the **AOW** thin film are smaller than those for the spin-coated thin film of **PIIDTT** (see Table S1 below). These GIWAXS data again indicate that thin film crystallinity is improved with the **AOW** approach. Moreover, the polymeric chains are predominately arranged in the *edge-on* mode on the substrate, while both *face-on* and *edge-on* packing modes coexist within the spin-coated thin film.

2.3. Chemical structure, AFM images and GIWAXS patterns of P3HT thin film

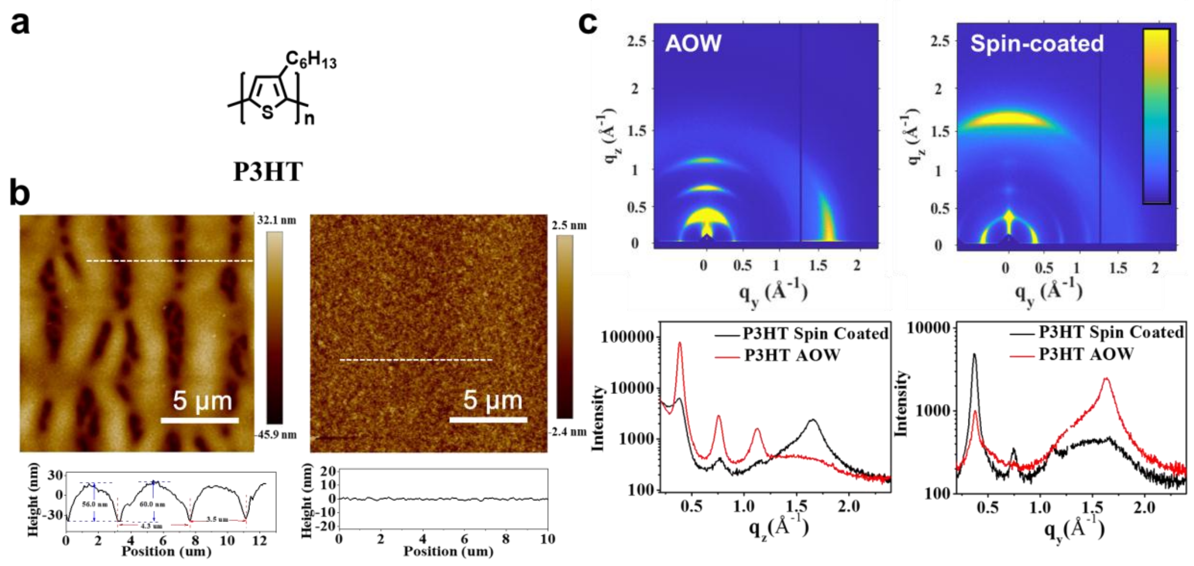

Figure S3. Chemical structure, AFM images and GIWAXS patterns of **P3HT** thin film. **a**, Chemical structure of **P3HT**; **b**, AFM height image of **P3HT** thin film with **AOW** method (*left*) and spin-coated film (*right*) with line-cuts (*below*); **c**, 2D GIWAXS patterns of **P3HT** thin film prepared with the **AOW** method (*left*) and spin-coated technique (*right*), and line-cuts (*below*) at q_z (*left*) and q_y directions (*right*).

Thin films of **P3HT** prepared with **AOW** and spin-coated approach show entirely different packing mode. The **AOW**-based thin film shows scattering signals at q_z = 0.39, 0.77, 1.12 Å^{-1} (owing to the lamellar stacking of alkyl chains) and at q_y = 1.64 Å^{-1} (owing to the interchain π-π stacking), thus polymeric chains adopt *edge-on* packing mode. However, polymeric chains of **P3HT** are packed dominantly with the *face-on* mode for the spin-coated thin film which displays lamellar stacking signals at q_y = 0.37, 0.74, 1.11 Å^{-1} and interchain π-π stacking signal at q_z = 1.66 Å^{-1}.

2.4 Chemical structure, AFM images and GIWAXS patterns of IDTBT thin film

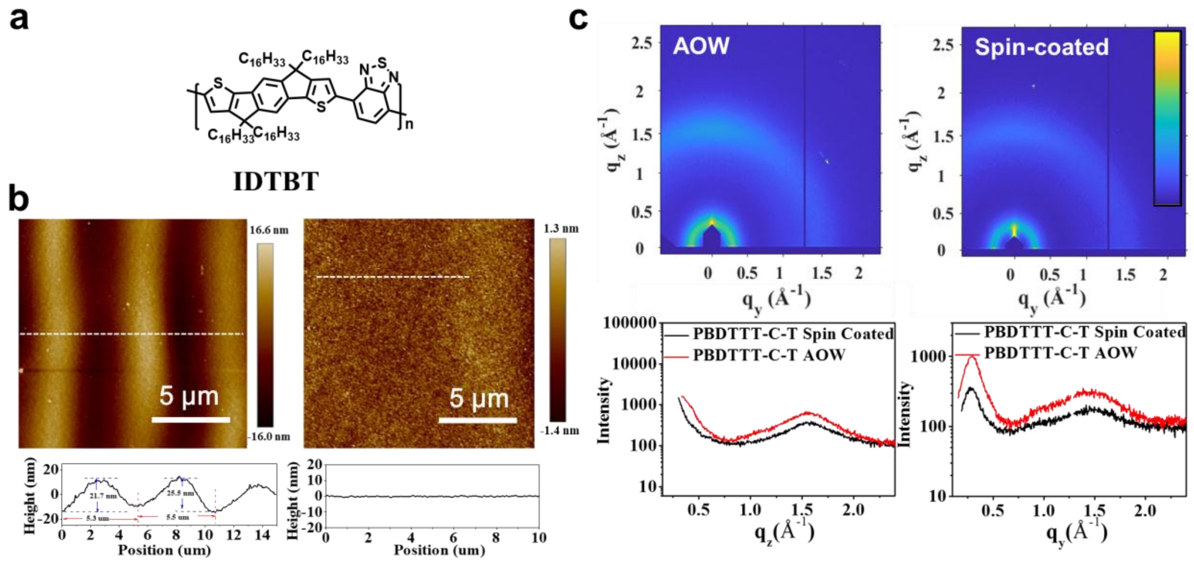

Figure S4. Chemical structure, AFM images and GIWAXS patterns of **IDTBT** thin film. **a**, Chemical structure of **IDTBT**; **b**, AFM height image of **IDTBT** thin film with **AOW** method (*left*) and spin-coated film (*right*) with line-cuts (*below*); **c**, 2D GIWAXS patterns of **IDTBT** thin film prepared with the **AOW** method (*left*) and spin-coated technique (*right*), and line-cuts (*below*) at q_z (*left*) and q_y directions (*right*).

2.5. Chemical structure, AFM images and GIWAXS patterns of PBDTTT-C-T thin film

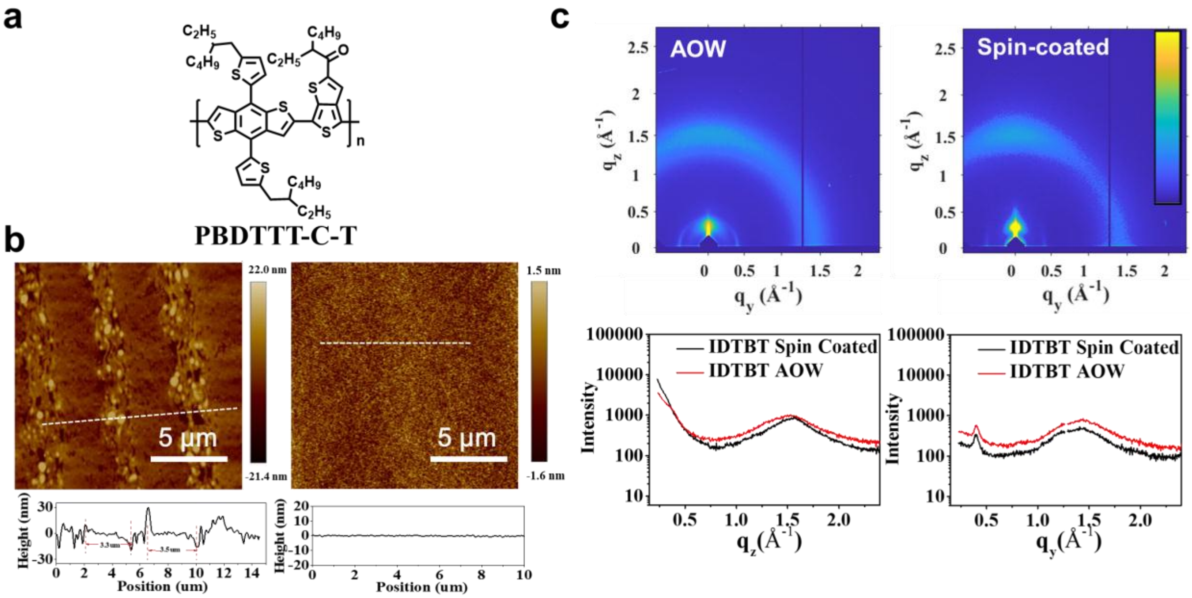

Figure S5. Chemical structure, AFM images and GIWAXS patterns of **PBDTTT-C-T** thin film. **a**, Chemical structure of **PBDTTT-C-T**; **b**, AFM height image of **PBDTTT-C-T** thin film with **AOW** method (*left*) and spin-coated film (right); **c**, 2D GIWAXS patterns of **PBDTTT-C-T** thin film prepared with the **AOW** method (*left*) and spin-coated technique (*right*), and line-cuts (*below*) at q_z (*left*) and q_y directions (*right*).

2.6. FWHM of GIWAXS signals for polymer thin films prepared with AOW and spin-coated methods

Table S1. Full width-at-half-maximum (FWHM) of GIWAXS signals for polymer thin films prepared with **AOW** and spin-coated methods.

Polymer		*in-plane* FWHM at q_y direction (Å^{-1})	*out-of-plane* FWHM at q_z direction (Å^{-1})				
		π-π stacking	lamella packing (X00)				
		(010)	(100)	(200)	(300)	(400)	(500)
**PDPP4T**	**AOW**	**0.180**	**0.037**	**0.055**	**0.077**	**0.131**	--
	Spin-coating	--	0.060	0.086	0.102	0.164	--
**PDPPTT**	**AOW**	**0.222**	**0.036**	**0.054**	**0.074**	**0.119**	--
	Spin-coating	0.257	0.075	0.128	0.130	--	--
**PIIDTT**	**AOW**	**0.090**	**0.027**	**0.035**	**0.048**	**0.065**	**0.079**
	Spin-coating	--	0.051	0.065	0.068	0.087	--
**P3HT**	**AOW**	**0.161**	**0.039**	**0.061**	**0.085**	--	--
	Spin-coating	--	--	--	--	--	--
**IDTBT**	**AOW**	--	--	--	--	--	--
	Spin-coating	--	--	--	--	--	--
**PBDTTT-C-T**	**AOW**	--	--	--	--	--	--
	Spin-coating	--	--	--	--	--	--

3. Characterizations of PDPP4T thin films by using AOW and other methods under different conditions

3.1. Characterizaions of AOW thin films of PDPP4T prepared at different conditions

3.1.1. Solvent effect

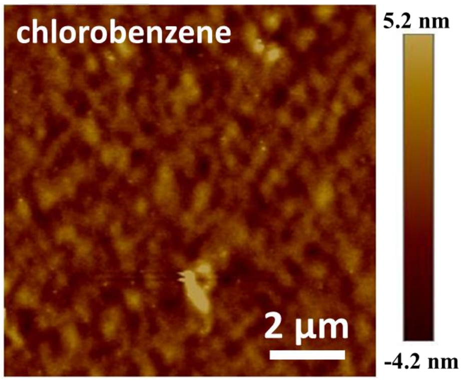

Figure S6. AFM height image of **PDPP4T** thin film prepared with the **AOW** method by using chlorobenzene as polymer solvent.

3.1.2. Temperature effect

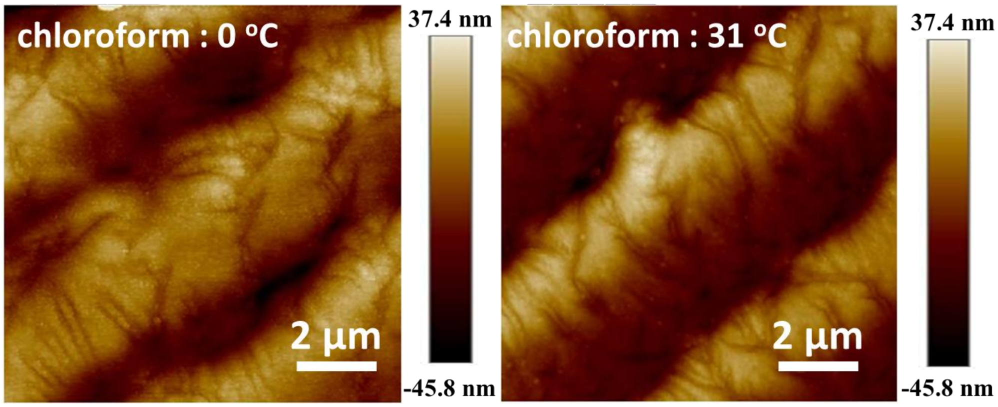

Figure S7. AFM height images of **PDPP4T** thin film prepared with the **AOW** method by using chloroform as polymer solvent at 0 ^{o}C (*left*) and 31 ^{o}C (*right*).

3.1.3. Substrate effect

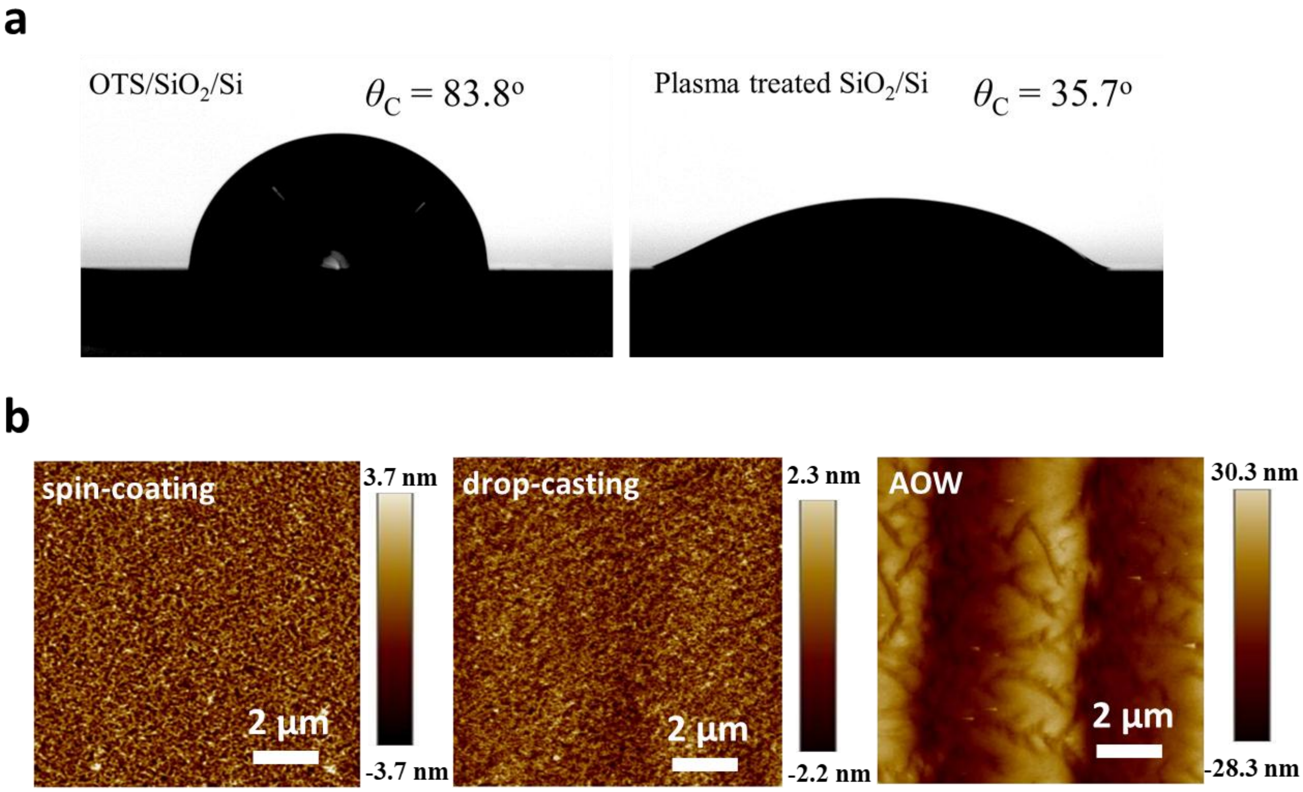

Figure S8. **a**, Contact angle of OTS-modified SiO_2/Si (*left*) and plasma treated SiO_2/Si (*right*) substrates; **b**, AFM height image of **PDPP4T** polymer thin film prepared on plasma treated SiO_2/Si substrate with spin-coating (*left*), drop-casting (*middle*) and **AOW** (*right*) method.

3.2. Characterizaions of thin films of PDPP4T by using other methods

3.2.1. Drop-casting method

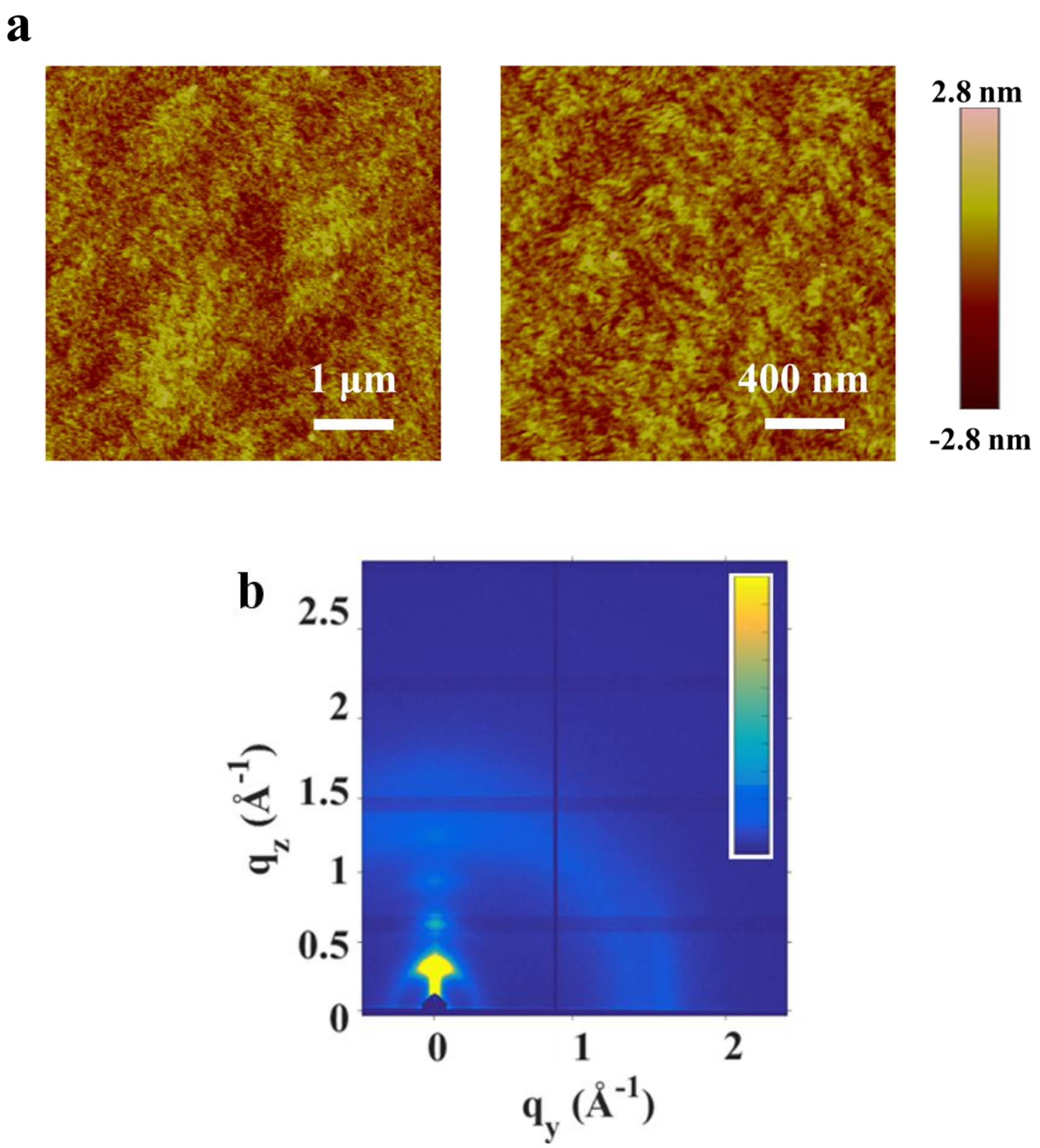

Figure S9. AFM image and GIWAXS pattern of **PDPP4T** thin film prepared with drop-casting method on OTS/SiO_2/Si solid substrate.

3.2.2. LS method

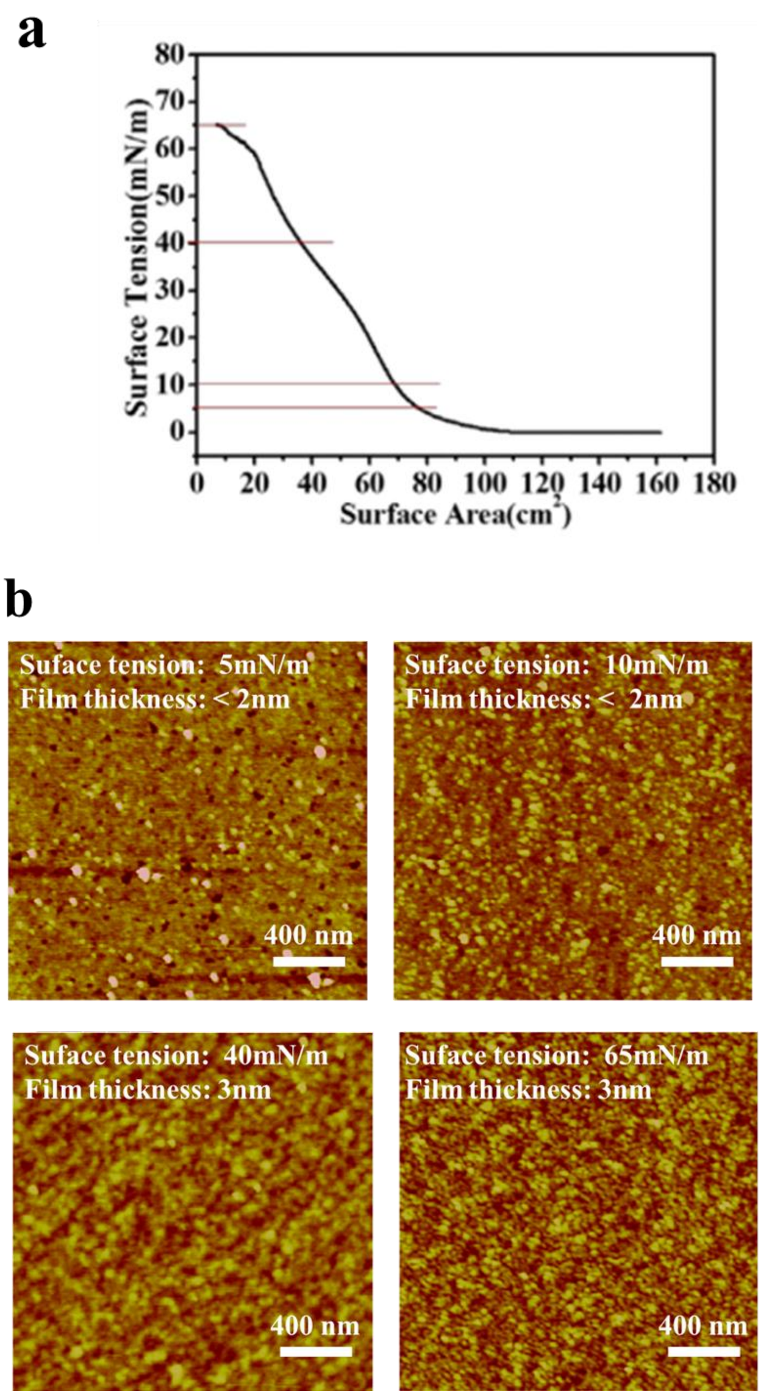

c

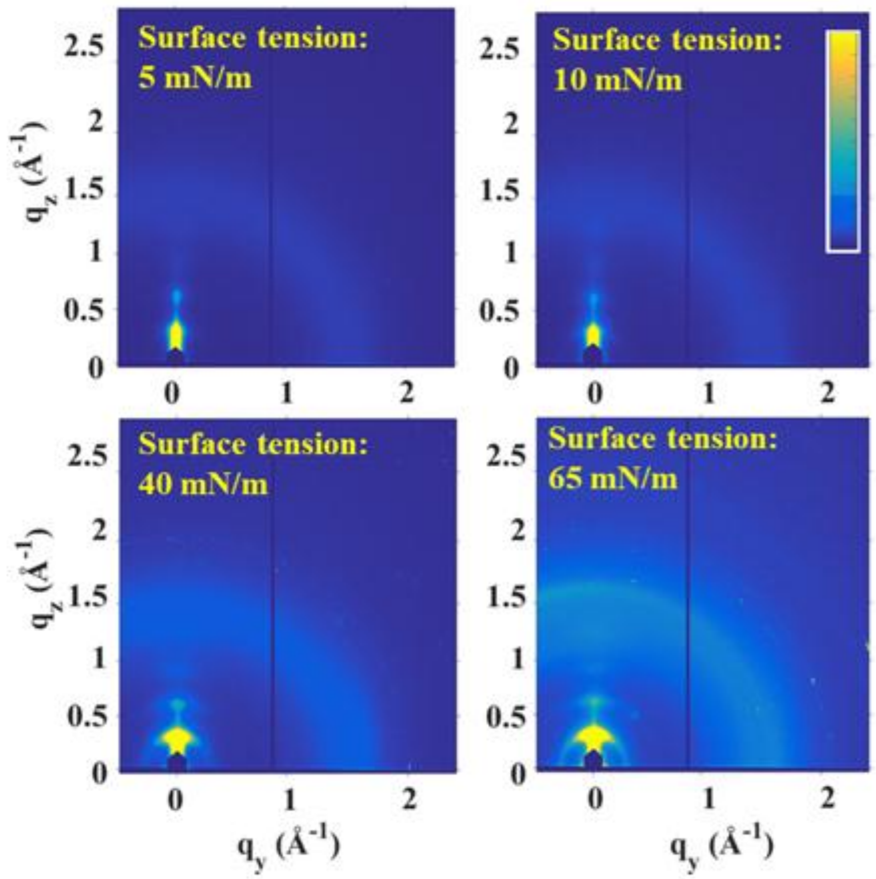

Figure S10. a) Surface pressure (π) versus mean monomeric area isotherm of **PDPP4T** spread from the $CHCl_3$ solution (0.1 mg mL^{-1}); b) AFM images of thin films prepared with LS method at different surface tensions, and c) GIWAXS patterns of thin films prepared with LS method at different surface tensions.

4. Fabrications and characterizations of FET devices and corresponding semiconducting data

4.1. FET device fabrication and characterization method

Bottom-gate bottom-contact organic field effect transistor devices were constructed on heavily *n*-doped silicon wafer with 300 nm thermally grown silicon oxide insulator (Capacitance=11.5 nF/cm^2). Gold electrodes with thickness of 28 nm were constructed on the SiO_2/Si substrate using photo-lithography techniques. Channel length (L) and width (W) are 50μm and 1400 μm, respectively. All substrates were used after OTS (octadecyltrichlorosilane) SAM modification. For OTS SAM modification process, wafers were initially cleaned in acetone and deionized water through sonication followed by 30 minutes immersion in piranha solution (mixture of 30% H_2O_2 and H_2SO_4 in 3/7 volume ratio), after which wafers were adequately washed by deionized water, isopropyl alcohol in sonication method. After drying by nitrogen flow, the surface was modified with n-octadecyltrichlorosilane (OTS). After that, OTS modified wafer was cleaned in chloroform, hexane, isopropyl alcohol. For spin-coated thin film technique, polymers need to be dissolved in solvents (**PDPP4T**, **IDTBT**, **PBDTTT-C-T**, **P3HT** in chloroform at room temperature, 5 mg/mL; **PDPPTT**, **PIIDTT** in anhydrous *o*-dichlorobenzene at 100 ^{o}C, 5 mg/mL). 100 μL of chloroform (or *o*-DCB) solution was spin coated at 3000 rpm for 40 s. The average thicknesses of spin-coated thin films were 74 nm for **PDPP4T**, 96 nm for **PDPPTT**, 59 nm for **PIIDTT**, 117 nm for **P3HT**, 88 nm for **IDTBT**, 66 nm for **PBDTTT-C-T**.

For the FET array fabrication, the OTS/SiO_2/Si substrate on which the drain-source gold electrodes were patterned in 8 × 12 matrix was utilized. The OTS SAM modification was conducted in the same way as depicted above. The **AOW** thin film of **PDPP4T** was prepared with a 50 μL solution of **PDPP4T** in chloroform (0.1 mg/mL). The substrate was inserted at an angle into the water to transfer the AOW thin film onto the substrate, and the resulting thin film was blown with dry nitrogen to remove residual water.

Electrical characterization of BGBC devices was conducted on Keithley 4200 Semiconductor Characterization System in ambient condition. The field-effect mobility in the saturation regime was extracted using the equation $I_{DS} = C_i\mu(V_{GS} - V_{Th})^2W/2L$, under the condition of $-V_{DS} > -(V_{GS} - V_{Th})$. And linear mobility was calculated according to equation $I_{DS} = (W/L)C_i\mu(V_{GS} - V_{Th})V_{DS}$, under condition of $-V_{DS} << -(V_{GS}-V_{Th})$, where I_{DS} is the source/drain current, μ is the field-effect mobility, W is the channel width, L is the channel length, C_i is the capacitance per unit area of gate dielectric layer, and V_{GS}, V_{Th}, and V_{DS} are the gate, threshold, and source/drain voltages, respectively.

4.2. FET characteriations of six polymers

4.2.1. Transfer and output characteristics of BGBC FETs based on polymer thin films

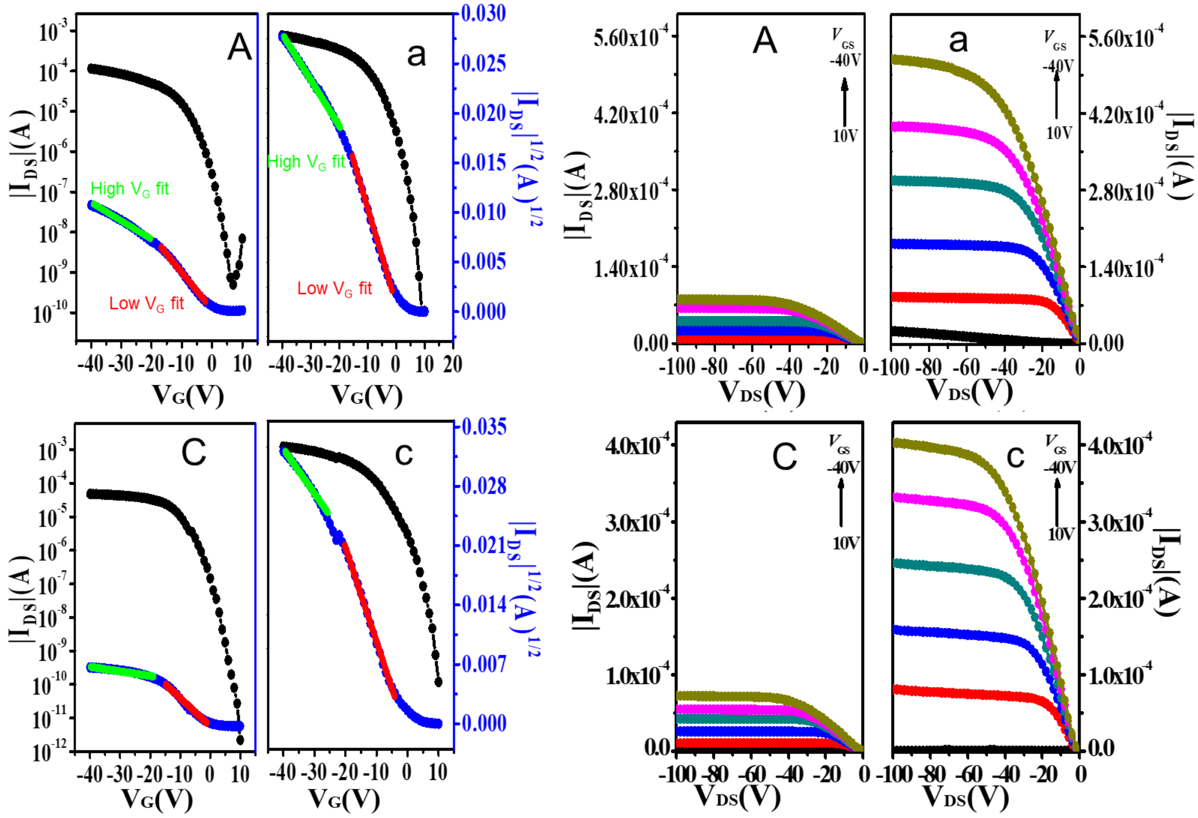

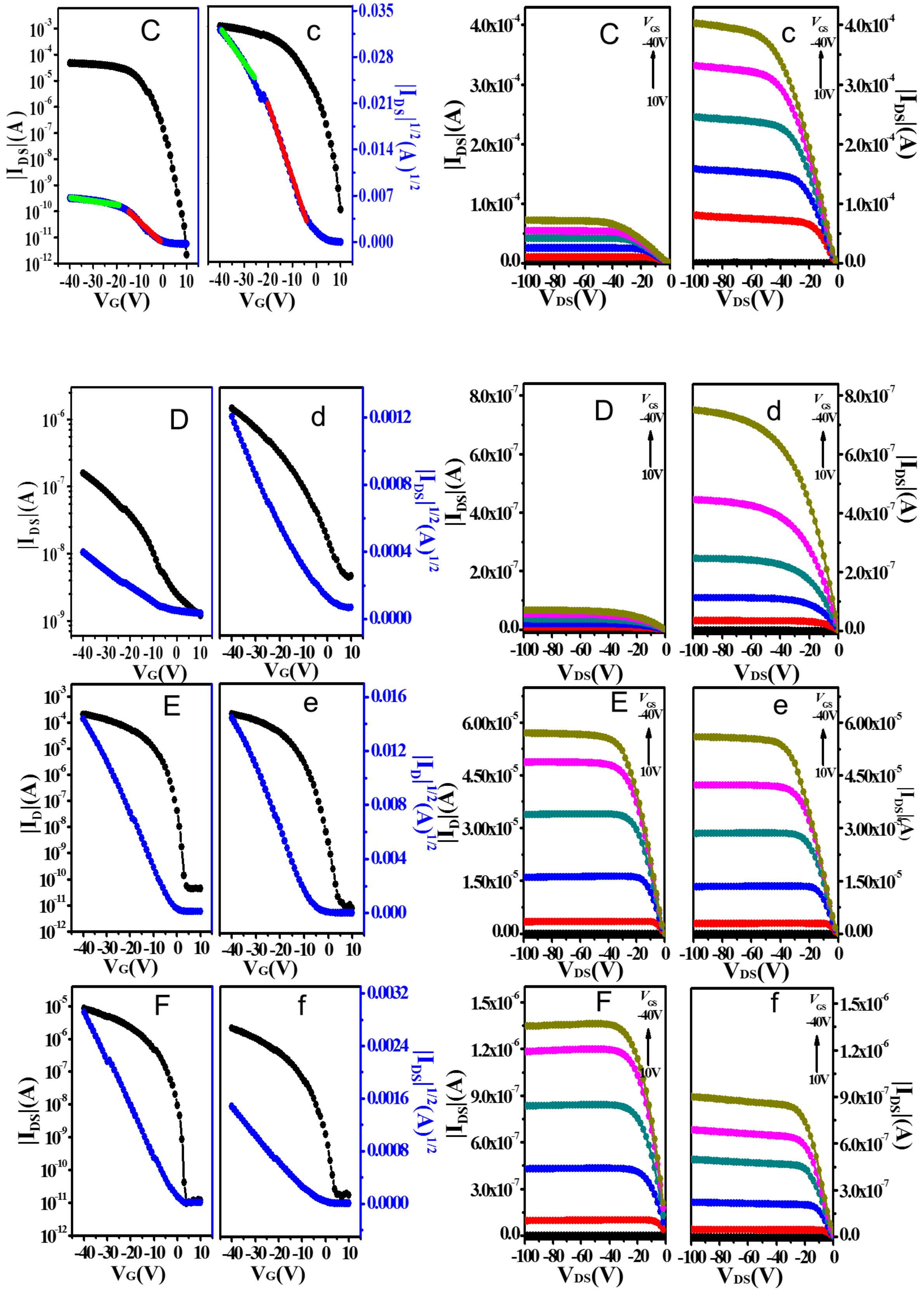

Figure S11. Transfer and output characteristics of BGBC FETs based on polymer thin film. **(A, a)**

PDPP4T, **(B**, **b) PDPPTT**, **(C**, **c) PIIDTT**, **(D**, **d) P3HT**, **(E**, **e) IDTBT, (F, f) PBDTTT-C-T. A-F** are for the spin-coated thin films. **a-f** are for thin films with **AOW** method, while **A-F** are for spin-coated thin films. The channel width (*W*) and length (*L*) are 1400 and 50μm, respectively. Mobilities of **PDPP4T**, **PDPPTT** and **PIIDTT** were extracted by fitting at high V_G (green) and low V_G (red) region.

4.2.2. Device stability of FET devices with AOW thin film of PDPP4T

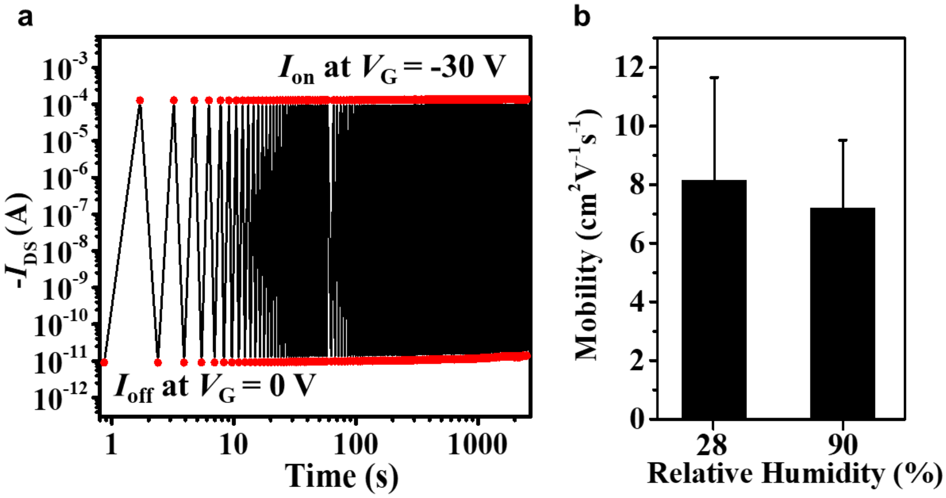

Figure S12. Device stability of FET devices based on **PDPP4T** thin films with AOW method. **a**, cyclic stability of a representative device (the V_G was switched between -30 V and 0 V repeatedly for 700 cycles); **b**, variation of hole mobility for FET device fabricated in different humidity conditions by using **AOW** method.

4.2.3. Hysteresis in transfer and output characteristics of thin film FETs of six polymers fabricated by AOW method

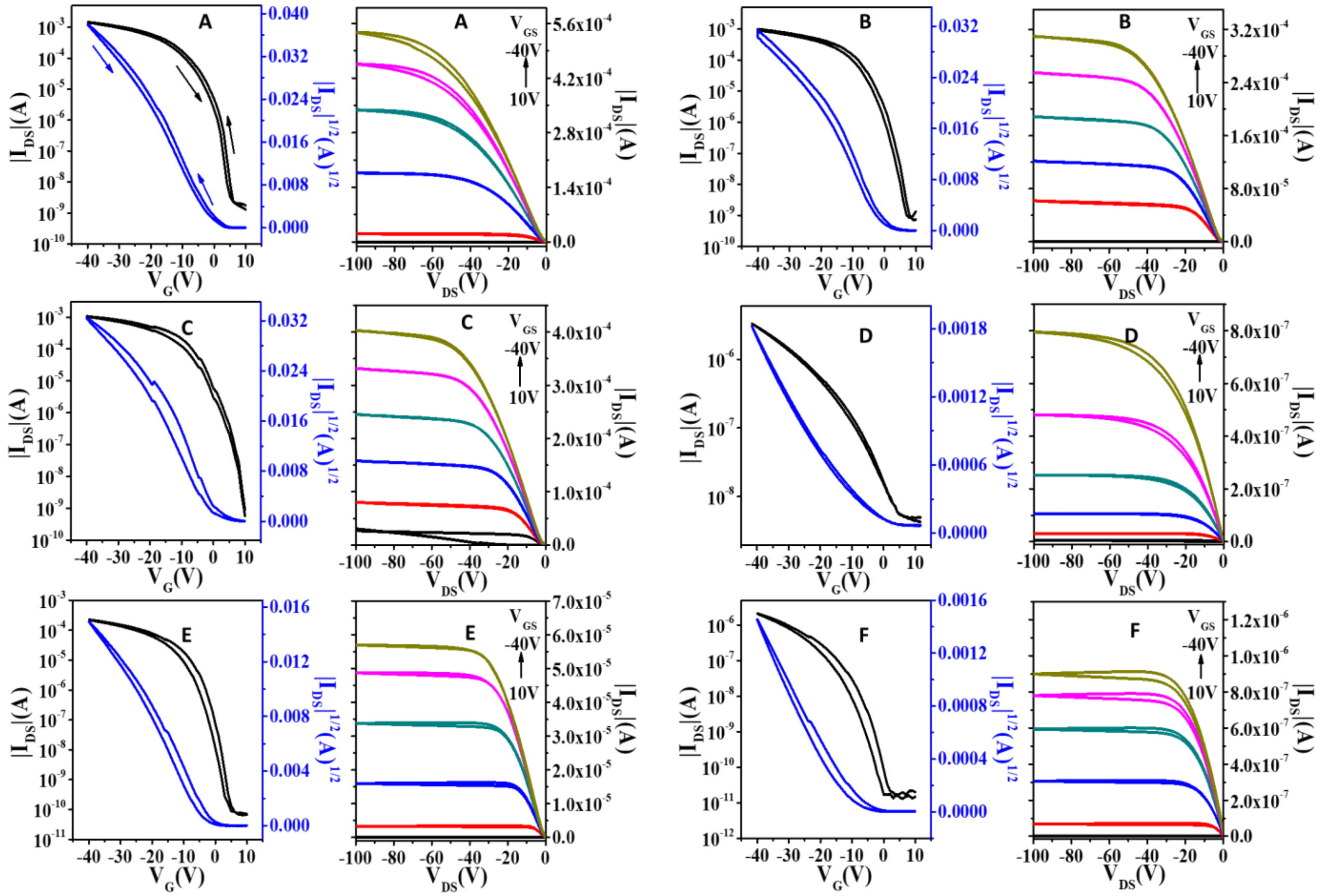

Figure S13. Hysteresis in transfer and output characteristics of thin film FETs of six polymers fabricated by **AOW** method. **(A) PDPP4T**, **(B) PDPPTT**, **(C) PIIDTT**, **(D) P3HT**, **(E) IDTBT, (F) PBDTTT-C-T.**

4.3. Charge mobilities of PDPP4T after thermal annealing

Table S2. Charge mobilities (in highest (average) form, extracted at low V_G region) of **PDPP4T** by using **AOW** or spin-coating methods before and after thermal annealing at 120 °C. All data were based on statistics of at least 20 BGBC FET devices with W = 1400 μm and L = 50 μm.

**PDPP4T**	**AOW** ($cm^2 V^{-1} s^{-1}$)	Spin-coating ($cm^2 V^{-1} s^{-1}$)	Reference[S3] ($cm^2 V^{-1} s^{-1}$)
Mobilities (R. T.)	11.66 (8.15)	1.05 (0.8)	--
Mobilities (120 °C)	13.46 (9.97)	3.20 (2.94)	5.50 (3.57)

5. Reference

[S1] J. Yao, C. Yu, Z. Liu, H. Luo, Y. Yang, G. Zhang, D. Zhang, *J. Am. Chem. Soc.* **2016**, *138*, 173.

[S2] T. Lei, Y. Cao, X. Zhou, Y. Peng, J. Bian, J. Pei, *Chem. Mater*. **2012**, *24*, 1762.

[S3] W. Hong, S. Chen, B. Sun, M. A. Arnould, Y. Meng, Y. Li, *Chem. Sci.* **2015**, *6*, 3225.
